# An enChIP system for the analysis of bacterial genome functions

**DOI:** 10.1186/s13104-018-3486-3

**Published:** 2018-06-14

**Authors:** Toshitsugu Fujita, Miyuki Yuno, Hodaka Fujii

**Affiliations:** 1 Department of Biochemistry and Genome Biology, Hirosaki University Graduate School of Medicine, 5 Zaifu-cho, Hirosaki, Aomori 036-8562 Japan; 2 Chromatin Biochemistry Research Group, Combined Program on Microbiology and Immunology, Research Institute for Microbial Diseases, Osaka University, 3-1 Yamadaoka, Suita, Osaka 565-0871 Japan

**Keywords:** enChIP, dCas9, ChIP, Chromatin immunoprecipitation, CRISPR, Bacteria

## Abstract

**Objective:**

The engineered DNA-binding molecule-mediated chromatin immunoprecipitation (enChIP) technology enables purification of specific genomic regions interacting with their associated molecules. In enChIP, the locus to be purified is first tagged with engineered DNA-binding molecules. An example of such engineered DNA-binding molecules to tag the locus of interest is the clustered regularly interspaced short palindromic repeats (CRISPR) system, consisting of a catalytically-inactive form of Cas9 (dCas9) and guide RNA (gRNA). Subsequently, the tagged locus is subjected to affinity purification for identification of interacting molecules. In our previous studies, we developed enChIP systems for analysis of mammalian genome functions. Here, we developed an enChIP system to analyze bacterial genome functions.

**Results:**

We generated a plasmid inducibly expressing *Streptococcus pyogenes* dCas9 fused to a 3xFLAG-tag (3xFLAG-dCas9) in bacteria. Inducible expression of 3xFLAG-dCas9 in *Escherichia coli* was confirmed by immunoblot analysis. We were able to purify specific genomic regions of *E. coli* preserving their molecular interactions. The system is potentially useful for analysis of interactions between specific genomic regions and their associated molecules in bacterial cells to understand genome functions such as transcription, DNA repair, and DNA recombination.

## Introduction

To understand the regulatory mechanisms underlying genome functions such as transcription, it is essential to identify the molecules associated with a genomic region of interest in vivo. The engineered DNA-binding molecule-mediated chromatin immunoprecipitation (enChIP) technology we developed recently, enables specific isolation of genomic regions of interest interacting with their associated molecules [1, 2]. Examples of such engineered DNA-binding molecules to tag the locus of interest are transcription activator-like proteins [3] and the clustered regularly interspaced short palindromic repeats (CRISPR) system [4, 5] consisting of a catalytically-inactive form of Cas9 (dCas9) and guide RNA (gRNA). Subsequently, the tagged locus is affinity-purified to identify interacting molecules. A locus of interest can be tagged in the cell by expressing engineered DNA-binding molecules [1, 2, 6–9] (in-cell enChIP) or in vitro using recombinant or synthetic engineered DNA-binding molecules [10, 11] (in vitro enChIP). After purification of the locus of interest, mass spectrometry (MS), RNA sequencing, and next-generation sequencing (NGS) can be used to identify proteins [1, 2, 6], RNAs [7], and genomic regions [9, 11] binding to the locus in a non-biased manner.

**Fig. 1:**
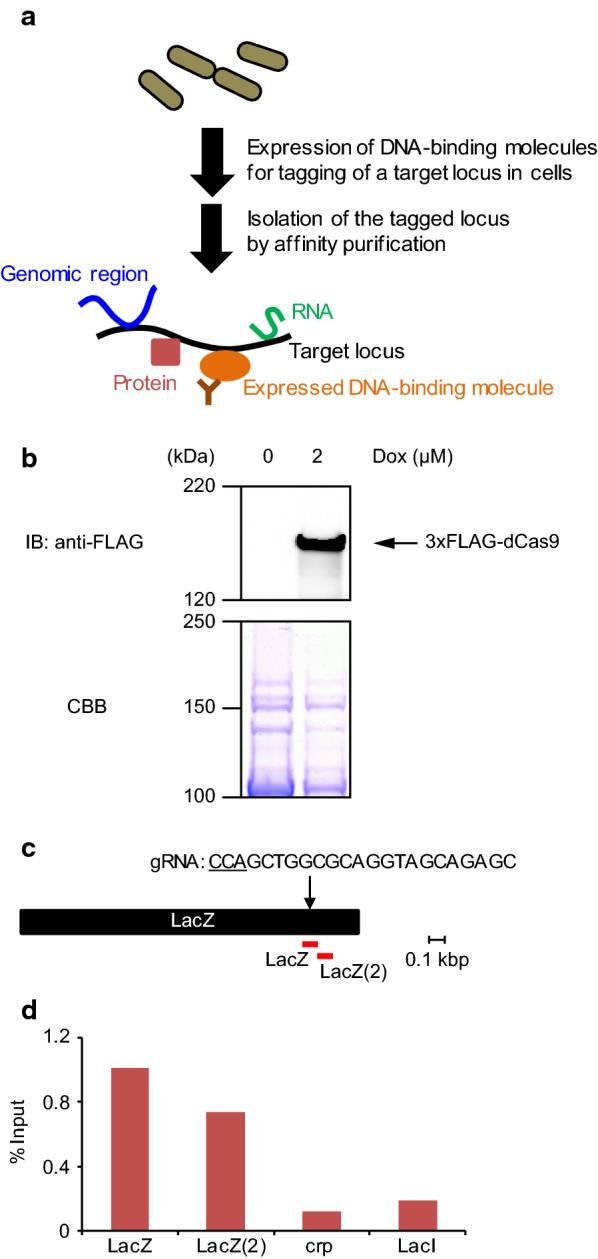
An enChIP system for biochemical analysis of bacterial genome functions. **a** Schematic of in-cell enChIP. Engineered DNA-binding molecules such as CRISPR can be used for locus tagging. The isolated materials can be subjected to biochemical analyses such as MS or NGS for identification of interacting molecules. **b** Inducible expression of 3xFLAG-dCas9. DH5α cells were transformed with 3xFLAG-dCas9/p-bacteria. The bacterial cells were incubated in the presence of doxycycline (Dox, 2 µM) for 4 h and subjected to immunoblot analysis with anti-FLAG M2 Ab. Coomassie brilliant blue (CBB) staining is shown as a protein loading control. **c** Positions of a gRNA and primer sets targeting the *lacZ* gene. The positions of primer sets are shown in red. The target sequence of the gRNA is shown, and the protospacer adjacent motif (PAM) sequence is underlined. **d** Isolation of the *lacZ* locus by enChIP. Real-time PCR analysis of chromatin complexes isolated by enChIP is shown. Irrelevant loci (*crp* and *lacI*) were analyzed as negative controls

Previously, we developed enChIP systems for analysis of mammalian genome functions. Here, we report development of an in-cell enChIP system for bacterial cells (Fig. 1a). The system consists of plasmids expressing *Streptococcus pyogenes* dCas9 fused to a 3xFLAG-tag (3xFLAG-dCas9) and gRNA in bacteria. The developed enChIP system isolated target genomic regions from *Escherichia coli*. The system might enable identification of molecules associated with a specific genomic region in bacteria, and thus help to elucidate their genome functions.

## Main text

### Materials and methods

#### Plasmids

To construct the doxycycline (Dox)-inducible 3xFLAG-dCas9 expression plasmid 3xFLAG-dCas9/p-bacteria (Addgene #64325), pdCas9-bacteria (Addgene #44249) [12] was digested with *Bgl*II and *Bst*Z17I and treated with bacterial alkaline phosphatase (*E. coli* C75) (2120A, Takara-Bio). The double-strand DNA (dsDNA) containing the coding sequence of the 3xFLAG-tag and the N-terminal portion of dCas9 (agatctaaagaggagaaaggatctATGGACTACAAAGACCATGACGGTGATTATAAAGATCATGACATCGATTACAAGGATGACGATGACAAGCTC*ATGGATAAGAAATACTCAATAGGCTTAGCTATCGGCACAAATAGCGTCGGATGGGCGGTGATCACTGATGAATATAAGGTTCCGTCTAAAAAGTTCAAGGTTCTGGGAAATACAGACCGCCACAGTATCAAAAAAAATCTTATAGGGGCTCTTTTATTTGACAGTGGAGAGACAGCGGAAGCGACTCGTCTCAAACGGACAGCTCGTAGAAG*GTATAC) (underlined: 3xFLAG-tag; italic: dCas9) was synthesized (Invitrogen) and digested with *Bgl*II and *Bst*Z17I. The cleaved pdCas9-bacteria and the synthetic DNA fragment were purified by agarose gel electrophoresis and ligated.

To construct vectors for expression of gRNAs, pgRNA-bacteria (Addgene #44251) [12] was digested with *Spe*I and *Hin*dIII and treated with bacterial alkaline phosphatase (*E. coli* C75). The dsDNA for targeting the *lacZ* gene (ACTAGTGCTCTGCTACCTGCGCCAGCGTTTTAGAGCTAGAAATAGCAAGTTAAAATAAGGCTAGTCCGTTATCAACTTGAAAAAGTGGCACCGAGTCGGTGCTTTTTTTGAAGCTT) (underlined: the target sequences) was synthesized (Invitrogen) and digested with *Spe*I and *Hin*dIII. The cleaved pgRNA-bacteria and the synthetic DNA fragment were purified by agarose gel electrophoresis and ligated. For construction of gRNAs targeting the promoter region of the *rpoH* gene, oligodeoxyribonucleotides (Table 1) were synthesized, annealed, and phosphorylated. Combination of oligodeoxyribonucleotides was; *E. coli* gRNA common: 27816 + 27817; rpoH p 158–180: 27818 + 27819; rpoH p 184–206: 27820 + 27821. The annealed dsDNAs were subjected to ligation reactions with the *Spe*I and *Hin*dIII-digested pgRNA-bacteria plasmid.

**Table 1:** Oligodeoxyribonucleotides used in this study

Number	Name	Sequence (5′ → 3′)	Experiments
27816	*E. coli* gRNA common S	agttaaaataaggctagtccgttatcaacttgaaaaagtggcaccgagtcggtgctttttttga	Construction of plasmids targeting the *rpo*H gene promoter
27817	*E. coli* gRNA common A	agcttcaaaaaaagcaccgactcggtgccactttttcaagttgataacggactagcct	Construction of plasmids targeting the *rpo*H gene promoter
27818	rpoH p 158–180 S	ctagtgttatactctttccctgcaagttttagagctagaaatagca	Construction of the plasmid targeting the *rpo*H gene promoter 158–180
27819	rpoH p 158–180 A	tattttaacttgctatttctagctctaaaacttgcagggaaagagtataaca	Construction of the plasmid targeting the *rpo*H gene promoter 158–180
27820	rpoH p 184–206 S	ctagtcggggtctctttccctgctagttttagagctagaaatagca	Construction of the plasmid targeting the *rpo*H gene promoter 184–206
27821	rpoH p 184–206 A	tattttaacttgctatttctagctctaaaactagcagggaaagagaccccga	Construction of the plasmid targeting the *rpo*H gene promoter 184–206
27792	LacZ-*E. coli*-F	gcgattaccgttgatgttgaagt	Real-time PCR in Figs. 1d and 2d (LacZ)
27793	LacZ-*E. coli*-R	agtaaggcggtcgggatagtttt	Real-time PCR in Figs. 1d and 2d (LacZ)
27794	LacZ-*E. coli*-F2	aaaactatcccgaccgccttact	Real-time PCR in Fig. 1d (LacZ(2))
27795	LacZ-*E. coli*-R2	gggaagacgtacggggtatacat	Real-time PCR in Fig. 1d (LacZ(2))
27796	crp-*E. coli*-F	tcacttcagagaaagtgggcaac	Real-time PCR in Fig. 1d (crp)
27797	crp-*E. coli*-R	gtcatagcgtctggttgttttgc	Real-time PCR in Fig. 1d (crp)
27798	LacI-*E. coli*-F	cgtcagtgggctgatcattaact	Real-time PCR in Fig. 1d (LacI)
27799	LacI-*E. coli*-R	atcaagaaataacgccggaacat	Real-time PCR in Fig. 1d (LacI)
27902	rpoH-*E. coli*-F	aagcttgcattgaacttgtggat	Real-time PCR in Fig. 2d (rpoH-prom)
27903	rpoH-*E. coli*-R	tatcttctggcgcttcagtggta	Real-time PCR in Fig. 2d (rpoH-prom)
27896	rpoH-coding-F	tacgttctgcgtaactggcgtat	Real-time PCR in Fig. 2d (rpoH-cod)
27897	rpoH-coding-R	accatttcgacttcatcctggtt	Real-time PCR in Fig. 2d (rpoH-cod)

#### *E. coli* strains

DH5α (9057, Takara-Bio) was transformed with 3xFLAG-dCas9/p-bacteria alone or together with gRNA expression plasmids, and transformed bacteria were selected with chloramphenicol (Cam) (25 µg/ml) for 3xFLAG-dCas9/p-bacteria alone or a combination of Cam (25 µg/ml) and ampicillin (Amp) (50 µg/ml) for 3xFLAG-dCas9/p-bacteria plus gRNA expression plasmid.

### Confirmation of inducible expression of 3xFLAG-dCas9

DH5α transformed with 3xFLAG-dCas9/p-bacteria was cultured in 2 ml of LB media containing Cam (25 µg/ml) at 37 °C overnight with shaking. One hundred microliters of the culture liquid was mixed with 900 µl of LB media containing Cam (25 µg/ml) and incubated for 1 h with shaking. Subsequently, Dox (2 µM) was added to the culture media for induction of expression of 3xFLAG-dCas9. After incubation with shaking for 4 h, 400 µl of the culture liquid was centrifuged at 5000 rpm for 1 min, and the bacterial pellets were suspended in 100 µl of 4× SDS buffer. After boiling at 100 °C for 5 min, 10 µl of the sample was subjected to SDS-PAGE with a 5–20% gradient gel followed by immunoblot analysis with anti-FLAG M2 antibody (Ab) (F1804, Sigma-Aldrich).

### enChIP-real-time PCR

DH5α cells transformed with 3xFLAG-dCas9/p-bacteria alone or together with a gRNA expression plasmid were cultured in 5 ml of LB media containing Cam (25 µg/ml) (3xFLAG-dCas9/p-bacteria) or Cam (25 µg/ml) plus Amp (50 µg/ml) (3xFLAG-dCas9/p-bacteria and a gRNA expression plasmid) overnight at 37 °C with shaking. The bacterial culture was added to 100 ml of LB media containing the same antibiotics and cultured at 37 °C with shaking. When OD_600_ of the culture media reached 0.5, Dox (2 µM) was added for induction of 3xFLAG-dCas9 expression. After incubation at 37 °C for 4.5 h with shaking, the bacterial cells were fixed with 1% formaldehyde at 37 °C for 5 min and neutralized with glycine at room temperature for 10 min. After centrifugation, the cell pellets were suspended in 800 µl of modified lysis buffer 3 [10 mM Tris, pH 8.0, 1 mM EDTA, 0.5 mM EGTA, 150 mM NaCl, 0.1% sodium deoxycholate, 0.1% SDS, and 1 × protease inhibitors (Complete-Mini, EDTA-free, 11836170001, Roche)], and DNA was fragmented by sonication using Ultrasonic disruptor UD-201 (TOMY SEIKO) with conditions: Output, 3; Duty, 100% (continuous); Time, Free; 6 cycles of sonication for 10 s and cooling on ice for 20 s (the average length of chromatin fragments was 1 kbp). The sonicated chromatin was subjected to enChIP-real-time PCR analysis as described previously [1, 2]. Primers used in the analysis are shown in Table 1.

## Results and discussion

### An enChIP system for the locus-specific biochemical analysis of bacterial genome functions

To analyze the locus-specific functions of the bacterial genome biochemically, we constructed a bacterial expression plasmid (3xFLAG-dCas9/p-bacteria (Addgene #64325)) encoding *S. pyogenes* dCas9 fused with the 3xFLAG-tag. The *E. coli* DH5α strain was transformed with 3xFLAG-dCas9/p-bacteria, and inducible expression of 3xFLAG-dCas9 by Dox was confirmed by immunoblot analysis with anti-FLAG Ab (Fig. 1b).

Next, for enChIP analysis, we designed a gRNA targeting the coding region of the *lacZ* gene (Fig. 1c). We transformed DH5α with 3xFLAG-dCas9/p-bacteria together with a gRNA expression plasmid targeting the *lacZ* gene in the DH5α genome. Four hours after the addition of Dox (2 µM), cells were crosslinked with formaldehyde and subjected to sonication for fragmentation of chromatin DNA. Next, chromatin complexes containing 3xFLAG-dCas9/gRNA were immunoprecipitated with anti-FLAG Ab, and DNA was purified. Real-time PCR showed enrichment of the *lacZ* gene (Fig. 1c, d). The percentage of input of negative control genomic regions (the *crp* and *lacI* genes) was markedly lower than that of the *lacZ* gene (Fig. 1d). These results showed that the bacterial enChIP system can specifically and efficiently isolate target genomic regions.

### enChIP analysis of an endogenous gene promoter

**Fig. 2:**
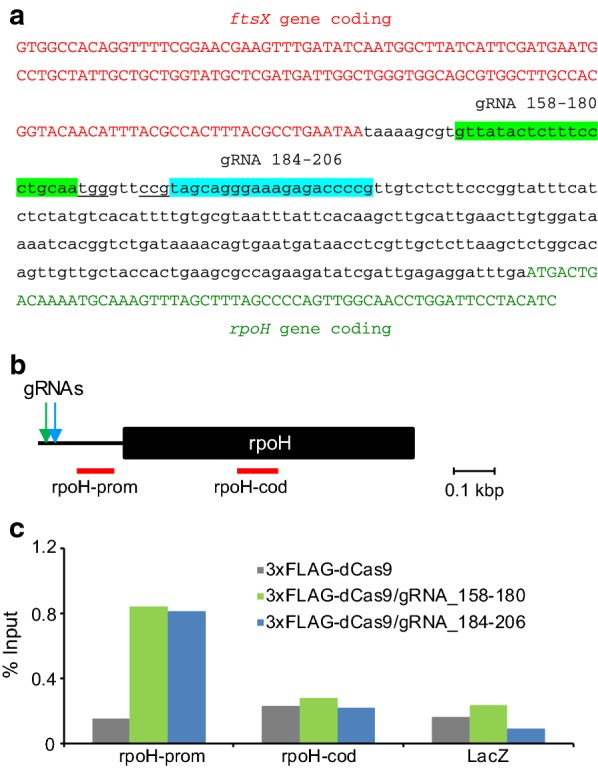
Isolation of an endogenous gene promoter by enChIP. **a** Positions of gRNAs in the *rpoH* promoter. Green highlight: gRNA 158–180; blue highlight: gRNA 184–206; red letters: *ftsX* gene coding sequence; green letters: *rpoH* gene coding sequence; underlined: protospacer adjacent motif (PAM) sequences. **b** Positions of gRNAs and primer sets targeting the *rpoH* gene. The positions of primer sets are shown in red. **c** Isolation of the *rpoH* gene promoter by enChIP. Real-time PCR analysis of chromatin complexes isolated by enChIP is shown. An irrelevant locus (*lacZ*) was analyzed as a negative control

Next, we targeted the promoter region of an endogenous gene, *rpoH*, which encodes RNA polymerase sigma (32) factor [13]. This gene is a single operon gene and one of the essential genes of *E. coli* [Profiling of *E. coli* Chromosome (PEC): https://shigen.lab.nig.ac.jp/ecoli/pec/]. First, we designed two gRNAs targeting the promoter region of the *rpoH* gene (Fig. 2a). Next, we transformed DH5α with 3xFLAG-dCas9/p-bacteria alone or together with one of two gRNA expression plasmids targeting the promoter region. After enChIP, real-time PCR showed that the promoter region of the *rpoH* gene was specifically enriched only in the immunoprecipitants prepared from DH5α cells transformed with both 3xFLAG-dCas9/p-bacteria and either one of two gRNA expression plasmids, but not from those transformed with 3xFLAG-dCas9/p-bacteria alone (Fig. 2b, c). The percentage of input of negative control genomic regions (the coding region of the *rpoH* gene or the *lacZ* gene) was markedly lower than that of the *rpoH* promoter region (Fig. 2b, c). These results confirmed that the bacterial enChIP system can specifically and efficiently isolate endogenous target genomic regions.

## Conclusions

In this study, we developed an enChIP system for analysis of bacterial genomes. This system enables efficient isolation of specific genomic regions from the *E. coli* genome while preserving their chromatin structures, and potentially contributes to the understanding of bacterial genome functions such as transcription and DNA repair.

## Limitations

Further studies might be necessary to assess the utility of this system combined with MS and NGS to identify molecules associated with the target genomic regions in bacteria.

## Abbreviations

enChIP: engineered DNA-binding molecule-mediated chromatin immunoprecipitation
CRISPR: clustered regularly interspaced short palindromic repeats
dCas9: a catalytically-inactive form of Cas9
gRNA: guide RNA
NLS: nuclear localization signal
MS: mass spectrometry
NGS: next-generation sequencing
Cam: chloramphenicol
Amp: ampicillin
Ab: antibody
